# Hospital- and patient-related factors associated with differences in hospital antibiotic use: analysis of national surveillance results

**DOI:** 10.1186/s13756-014-0040-5

**Published:** 2014-12-24

**Authors:** Jon Birger Haug, Dag Berild, Mette Walberg, Åsmund Reikvam

**Affiliations:** Department of Infectious Diseases, Oslo University Hospital Trust, Oslo, Norway; Microbiology Section, Laboratory Centre, Vestre Viken Hospital Trust, Drammen, Norway; Institute of Clinical Medicine, Faculty of Medicine, University of Oslo, Oslo, Norway; Department of Pharmacology, Oslo University Hospital Trust, Oslo, Norway

**Keywords:** Antibiotic use, Antibiotic surveillance, Hospitals, Risk factors

## Abstract

**Background:**

Surveillance data of antibiotic use are increasingly being used for benchmarking purposes, but there is a lack of studies dealing with how hospital- and patient-related factors affect antibiotic utilization in hospitals. Our objective was to identify factors that may contribute to differences in antibiotic use.

**Methods:**

Based on pharmacy sales data (2006–2011), use of all antibiotics, all penicillins, and broad-spectrum antibiotics was analysed in 22 Health Enterprises (HEs). Antibiotic utilization was measured in World Health Organisation defined daily doses (DDDs) and hospital-adjusted (ha)DDDs, each related to the number of bed days (BDs) and the number of discharges. For each HE, all clinical specialties were included and the aggregated data at the HE level constituted the basis for the analyses. Fourteen variables potentially associated with the observed antibiotic use – extracted from validated national databases – were examined in 12 multiple linear regression models, with four different measurement units: DDD/100 BDs, DDD/100 discharges, haDDD/100 BDs and haDDD/100 discharges.

**Results:**

Six variables were independently associated with antibiotic use, but with a variable pattern depending on the regression model. High levels of nurse staffing, high proportions of short (<2 days) and long (>10 days) hospital stays, infectious diseases being the main ICD-10 diagnostic codes, and surgical diagnosis-related groups were correlated with a high use of all antibiotics. University affiliated HEs had a lower level of antibiotic utilization than other institutions in eight of the 12 models, and carried a high explanatory strength. The use of broad-spectrum antibiotics correlated strongly with short and long hospital stays. There was a residual variance (30%–50% for all antibiotics; 60%–70% for broad-spectrum antibiotics) that our analysis did not explain.

**Conclusions:**

The factors associated with hospital antibiotic use were mostly non-modifiable. By adjusting for these factors, it will be easier to evaluate and understand observed differences in antibiotic use between hospitals. Consequently, the inter-hospital differences can be more confidently acted upon. The residual variation is presumed to largely reflect prescriber-related factors.

## Background

In working towards rational use of antibiotics in hospitals, one needs to establish and maintain a suitable system for surveillance of antibiotic use [1]. However, the surveillance commonly applied is hampered by methodological pitfalls that impede the interpretation of the surveillance findings [2].

First, antibiotic utilization measurement using the number of patient bed days (BDs) as denominator may give results and interpretations that differ from those obtained when the number of patient discharges is used. By applying both denominators, a better understanding of the temporal trends in antibiotic use can be gained [3-5].

Second, the World Health Organisation (WHO)-derived system of defined daily doses (DDDs), although internationally accepted as units of measurement for drug utilization, is not always suitable for showing antibiotic use in hospitalized patients because the WHO doses may differ from the recommended antibiotic doses or the doses that are actually prescribed [6,7]. Alternative units have been considered [8,9]. In a recent study, we found a marked difference between WHO defined doses (WHO DDDs) and doses recommended in hospital guidelines, especially for the penicillins [10]. The discrepancy had consequences for the interpretation of the data on antibiotic use and we suggested that WHO DDDs should be supplemented with hospital-adjusted defined daily doses (haDDDs) in the surveillance of antibiotic use.

A further challenge in surveillance methodology is to identify factors that affect the use of antibiotics in hospitals. Few studies have addressed this issue. The aim of the present study was to investigate, by use of a national surveillance data set, the extent to which relevant, validated hospital- and patient-related variables can explain differences in antibiotic use.

## Methods

### Study hospitals (Health Enterprises, HEs)

We registered data on antibiotic use in the period from 2006 to 2011 (six years) for the 19 public HEs (five university-affiliated and 14 large general HEs) and three large private HEs in Norway. Each public HE consists of one to seven hospital units and covers a complete and comparable spectrum of specialties, except specialized units for transplantation, heart surgery, neurosurgery, burns and multitrauma that are established only at the university hospitals. The three private institutions include mainly general internal medicine and surgery and intensive care units. We excluded four private institutions with specialized functions for elective orthopaedics and rheumatology, cardiac surgery and rehabilitation, and all psychiatric and drug abuse institutions.

Ideally, analyses of antibiotic use should be performed at the level of hospital units, and the distribution of clinical specialties within each hospital should be known. However, at present administrative and clinical data of this kind are not routinely available from official and validated national sources. The lowest level at which this information may be acquired is the HE. Consequently, data on antibiotic use were analysed for whole HEs.

### Antibiotic use

We have previously reported the method for antibiotic data acquisition [10]. Briefly, we acquired data on hospital antibiotic use from a national pharmacy database. The data set was processed in a Microsoft Excel spreadsheet and further analysed in the statistical program Stata version 12 (StataCorp LP, College Station, TX). All systemic antibacterial agents except methenamine included in the Anatomical Therapeutic Chemical (ATC) DDD group J01 were registered. From other ATC DDD groups, we included oral vancomycin, rifampicin and oral metronidazole.

Data on antibiotic use was expressed in DDDs using the 2011 WHO ATC/DDD classification [11]. DDDs were related to length of stay, which was measured in BDs, defined as the date of discharge minus the admission date. The number of patient discharges was used as an additional denominator for measure of antibiotic use [12].

In a previous study, we adjusted the WHO DDDs for a number of antibiotic substances [10]. These DDDs, designated haDDDs, were based on dose recommendations outlined in regional and national antibiotic guidelines [13]. The same haDDD values supplemented the WHO DDDs in the current study.

### Dependent (outcome) variables

Total antibiotic use (“all antibiotics”) in the period 2006 to 2011 for the 22 HEs was the main dependent variable in the regression analyses. We also designated two subgroups as dependent variables: use of “broad-spectrum antibiotics” (second- and third-generation cephalosporins, fluoroquinolones, carbapenems, and penicillins with enzyme inhibitors) and use of “all penicillins” (penicillinase sensitive, penicillinase resistant and extended-spectrum penicillins). Each of these three antibiotic groups was analysed using the following measurement units: DDD/100 BDs, DDD/100 discharges, haDDD/100 BDs and haDDD/100 discharges.

### Independent variables

Administrative data and candidate explanatory variables for each HE were derived from publicly available on-line databases maintained by Statistics Norway [14]. For the regression analyses, we included independent variables that were considered clinically plausible and thus possibly associated with antibiotic use. Moreover, we required the variables to be clearly defined, quality assessed by a recognized national body, and easily accessible. These requirements were set to establish a reproducible, robust data set of optimal quality.

The 11 continuous variables (Table 1) were: per cent of hospital stays lasting < 2 days, per cent of hospital stays lasting > 10 days, number of physicians per 100 hospital beds, number of registered nurses per 100 hospital beds, per cent of discharges with a cancer ICD-10 main diagnosis, per cent of discharges with an infectious diseases ICD-10 main diagnosis, per cent of discharges with a surgical main diagnosis-related group (DRG), per cent of discharges with a medical main DRG, number of day care treatments, number of ambulatory consultations for all patients and number of ambulatory consultations for patients with infectious diseases (the last three variables measured per 100 hospital beds). The variables for day care and ambulatory patients were included because these patients were given antibiotics from the same ward stock as the in-patients.

**Table 1:** Measurement units and value ranges for continuous variables entered into 12 linear regression models

**Continuous variables**	**Unit**	**Data point** ^**a**^ **range**	**Mean**
Hospital stay < 2 days	% of discharges	24.5–40.5	32.4
Hospital stay > 10 days	% of discharges	5.2–17.3	9.7
Number of physicians^b^	per 100 hospital beds	38.8–128.3	63.9
Number of nurses	per 100 hospital beds	132.5–300.1	197.6
ID^c^ main ICD-10 diagnosis	% of discharges	1.6–6.0	2.9
Cancer main ICD-10 diagnosis^b^	% of discharges	3.6–17.1	8.0
Surgical DRGs	% of discharges	18.0–39.8	27.5
Medical DRGs^b^	% of discharges	46.4–79.6	65.9
All ambulatory consultations^b^	per 100 hospital beds	89.9–543.3	327.6
ID^c^ ambulatory consultations^d^	per 100 hospital beds	0.5–7.7	2.7
Day-care treatments^d^	per 100 hospital beds	10.1–79.5	35.9

^a^132 data points: six years of 22 Health Enterprise's annual data.

^b^Variable removed from the regression models due to collinearity.

^c^ID: (any) infectious diseases.

^d^Variable included in model, but not significantly associated with antibiotic use.

Three categorical independent variables were also included (Table 2). These were university versus non-university affiliation, size of the HE (<300 hospital beds, 300–600 beds and > 600 beds) and geographical region (i.e. belonging to one of four Norwegian Health Regions).

**Table 2:** **Description of three categorical variables** ^**a**^ **entered into 12 linear regression models**

**Variables (No. of data points)**	**% of beds**	**% of bed days**	**% of discharges**	**Average HE stay (d)**
University HEs^b^ (30)	40.6	40.7	38.5	4.9
Non-university HEs (102)	59.4	59.3	61.5	4.5
Health Region 1 (60)	54.0	54.2	55.5	4.5
Health Region 2 (30)	20.3	20.8	20.5	4.7
Health Region 3 (18)	14.3	14.5	13.8	4.8
Health Region 4 (24)	11.4	10.4	10.2	4.7
HEs < 300 beds (35)	9.0	8.6	8.3	4.8
HEs 300–600 beds (50)	31.0	31.7	33.3	4.4
HEs > 600 beds (47)	60.0	59.7	58.5	4.7

132 data points (22 HEs^b^ over 6 years) for each independent variable.

^a^Of the three categorical variables, only university affiliation was independently associated with antibiotic use in eight of the 12 regression models.

^b^HE = Health Enterprise.

All HEs used the same DRG version based on the WHO ICD-10 classification (NordDRG, version NOR PR1) during the study period. A surgical main DRG denotes a hospital stay during which a procedure was performed in an operating theatre. A medical main DRG was registered when no such procedure took place.

### Statistical analyses

Collection of the annual data on antibiotic use for 22 HEs over six years resulted in data sets containing 132 observations. Analyses were done with Stata statistical software version 12 (StataCorp LP, College Station, TX). For correlations between continuous variables, Pearson correlations (Stata procedure: 'pwcorr') was used. Since our data were normally distributed and the dependent variables continuous, we analysed 12 different multiple linear regression models (procedure: 'regress'). The same 14 independent variables were introduced in all regression models.

To account for possible dependence of observations within the individual HEs, that is to say dependence related to repeated and possibly correlated annual measures for the 22 HEs, we performed robust linear regression analyses with HEs as clusters (variance estimator option 'cluster').

In a stepwise approach, a test for collinearity of the independent variables (i.e. the extent to which the variables are related to each other) was performed to fit the final model. We used the variance inflation factor (vif) which tests for multivariate multicollinearity (procedure: 'estat vif'). In each regression step, the variable was excluded that had the highest vif, i.e. for which the least amount of its variance was associated with the outcome. This was repeated until no variable had a vif > 5 [15].

Because of a relatively large number of independent variables, the adjusted R square (aR^2^) was calculated for each regression model to show how well it fitted the data. For all analyses, aR^2^ > 0.3 was considered a strong correlation. A two-tailed *P-*value < 0.05 was set as a limit for statistical significance. To assess the unique contribution of each independent variable to the increment in aR^2^ in the final models, a conservative semi-partial R^2^ was calculated for each variable using Stata procedure 'pcorr' [15].

## Results

### All antibiotic and broad-spectrum antibiotic use

**Figure 1:**
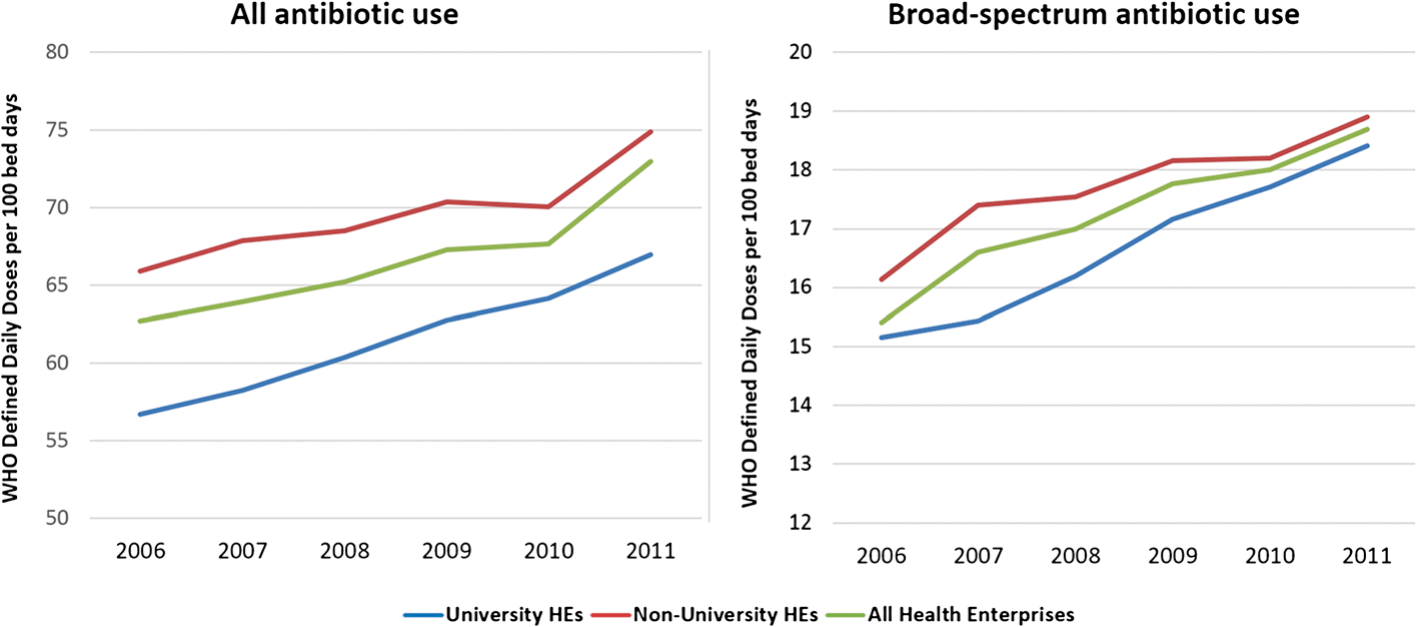
**All antibiotic and broad-spectrum antibiotic use in 22 Norwegian Health Enterprises (HEs), 2006 – 2011.** Annual utilization averages for all HEs and according to university affiliation of HEs.

From 2006 to 2011, the mean annual use, measured by WHO DDDs per 100 bed days, increased for “all antibiotics” from 62.7 to 73.0 and for “broad-spectrum antibiotics” (BSAs) from 15.4 to 18.7 (Figure 1). For each year a lower level was registered for university HEs than for non-university HEs, all specialties combined, with regard to both all antibiotic use and BSA use. However, for BSA use this difference diminished during the study period. For all antibiotic use and BSA use related to number of discharges no significant increases were found during the period (data not shown).

### Multivariate regression analyses

Fourteen independent variables were entered into the multiple linear regression models, of which four were removed from the analyses because of collinearity: medical DRGs, infectious diseases ambulatory consultations, cancer main ICD-10 diagnosis, and number of physicians. Of the remaining ten variables that were included in the models, six were found to be independently and significantly correlated with antibiotic use. A correlation was found for between two and five of these six variables depending on the outcome measure, i.e. one of the 12 combinations of antibiotic group and units of measurement (Tables 3 and 4).

**Table 3:** **Explanatory factors significantly related to antibiotic use (number of bed days as denominator) in Norwegian Health Enterprises, 2006–2011, derived from six multiple linear regression models**

**Independent variables**	**WHO DDDs/100 bed days**			**Hospital-adjusted DDDs/100 bed days**		
	**All antibiotics**	**Broad-spectrum** ^**a**^	**All penicillins** ^**b**^	**All antibiotics**	**Broad-spectrum** ^**a**^	**All penicillins** ^**b**^
**Overall model, R** ^**2**^ **(adjusted R** ^**2**^ **)**	**0.49 (0.43)**	**0.40 (0.33)**	**0.55 (0.50)**	**0.43 (0.37)**	**0.36 (0.30)**	**0.59 (0.54)**
**Infect. disease ICD-10 main diag. (%)**						
Regression coeff. (95% C.I.)	5.35^***^ (2.33; 8.38)		3.83^***^ (1.34; 6.33)	2.86^**^ (1.00; 4.71)		1.56^**^ (0.59; 2.54)
Beta weight	0.47		0.45	0.41		0.44
Increment in R^2^	0.0690		0.0633	0.0509		0.0606
**Registered nurses per 100 beds**						
Regression coeff. (95% C.I.)	0.17^***^ (0.08; 0.26)		0.10^***^ (0.04; 0.15)	0.10^**^ (0.04; 0.17)	0.02^*^ (0.001; 0.04)	0.04^**^ (0.02; 0.06)
Beta weight	0.56		0.44	0.57	0.28	0.39
Increment in R^2^	0.1141		0.0706	0.1163	0.0293	0.0548
**Hospital stay < 2 days (%)**						
Regression coeff. (95% confidence interval (C.I.))	0.81^*^ (0.04; 1.57)	0.31^*^ (0.05; 0.58)			0.29^*^ (0.04; 0.53)	
Beta weight	0.29	0.38			0.40	
Increment in R^2^	0.0422	0.0718			0.0829	
**University hospital (binary**)						
Regression coeff. (95% C.I.)	−11.82^**^ (−20.5; −3.2)	−2.65^***^ (−4.01; −1.29)		−8.22^***^ (−12.3; −4.1)	−2.72^***^ (−3.53; −1.91)	
Beta weight	−0.55	−0.41		−0.62	−0.49	
Increment in R^2^	0.1078	0.0607		0.1354	0.0868	
**Surgical DRG**s^c^ **per 100 beds**						
Regression coeff. (95% C.I.)		0.15^*^ (0.01; 0.30)		0.52^*^ (0.06; 0.97)		
Beta weight		0.29		0.48		
Increment in R^2^		0.0222		0.0589		

Significance levels: *P < 0.05, **P < 0.01, ***P < 0.001.

^a^Broad-spectrum antibiotics: second- and third-generation cephalosporins, fluoroquinolones, carbapenems, and penicillins with enzyme inhibitors.

^b^All penicillins: penicillinase sensitive, penicillinase resistant and extended-spectrum penicillins.

^c^DRG: Diagnosis-related groups.

**Table 4:** **Explanatory factors related to antibiotic use (number of discharges as denominator) in 22 Norwegian Health Enterprises, 2006–2011, derived from six multiple linear regression models**

**Independent variables**	**WHO DDDs/100 discharges**			**Hospital-adjusted DDDs/100 discharges**		
	**All antibiotics**	**Broad-spectrum** ^**a**^	**All penicillins** ^**b**^	**All antibiotics**	**Broad-spectrum** ^**a**^	**All penicillins** ^**b**^
**Overall model, R** ^**2**^ **(adjusted R** ^**2**^ **)**	**0.71 (0.67)**	**0.30 (0.22)**	**0.68 (0.65)**	**0.71 (0.68)**	**0.34 (0.26)**	**0.72 (0.69)**
**Infect. disease ICD-10 main diag. (%)**						
Regression coeff. (95% confidence interval (C.I.))	30.6^***^ (15.9; 45.2)		21.9^***^ (10.0; 33.8)	16.4^***^ (7.5; 25.4)		8.95^***^ (4.3; 13.6)
Beta weight	0.44		0.45	0.36		0.44
Increment in R^2^	0.0588		0.0614	0.0406		0.0591
**Registered nurses per 100 beds**						
Regression coeff. (95% C.I.)	0.78^***^ (0.37; 1.20)		0.46^***^ (0.21; 0.72)	0.49^***^ (0.21; 0.77)		0.17^**^ (0.07; 0.27)
Beta weight	0.43		0.36	0.42		0.43
Increment in R^2^	0.0657		0.0463	0.0625		0.0357
**Hospital stay > 10 days (%)**						
Regression coeff. (95% (C.I.)	12.1^**^ (5.1; 19.2)	2.38^**^ (0.9; 3.86)	7.46^*^ (1.2; 13.7)	8.27^***^ (4.4; 12.2)	2.75^**^ (1.2; 4.3)	2.99^***^ (0.4; 5.6)
Beta weight	0.48	0.45	0.42	0.51	0.56	0.41
Increment in R^2^	0.0861	0.0753	0.0663	0.0956	0.1157	0.0616
**University hospital (binary**)						
Regression coeff. (95% C.I.)	−47.6^***^ (−84.6; −10.7)	−9.8^**^ (−15.5; −4.1)		−33.6^***^ (−51.1; −16.2)	−10.9^***^ (−14.5; −7.2)	
Beta weight	−0.36	−0.35		−0.39	−0.42	
Increment in R^2^	0.0458	0.0441		0.0545	0.0626	
**Surgical DRGs** ^**c**^ **per 100 beds**						
Regression coeff. (95% C.I.)				2.43^*^ (0.2; 4.6)		
Beta weight				0.35		
Increment in R^2^				0.0316		

Significance levels: *P < 0.05, **P < 0.01, ***P < 0.001.

^a^Broad-spectrum antibiotics: second- and third-generation cephalosporins, fluoroquinolones, carbapenems, and penicillins with enzyme inhibitors.

^b^All penicillins: penicillinase sensitive, penicillinase resistant and extended-spectrum penicillins.

^c^DRG: Diagnosis-related groups.

We found that for all regression models, except for BSA use measured in WHO DDDs and haDDDs per 100 discharges, the aR^2^ equalled or significantly exceeded 0.3, which indicates that the overall models fitted the data well [15].

### Hospital characteristics and geographical area

Of the three categorical variables (Table 2), only a university affiliation of the HE was independently associated with antibiotic use (Tables 3 and 4). A university affiliation of the HE was strongly and negatively correlated with the use of all antibiotics and use of BSAs, both when BDs and discharges were used as denominator. No significant difference was found between university and non-university HEs with regard to penicillin use. The lower level of utilization in university HEs, measured in WHO DDDs/100 BDs, amounted to −11.8 for all antibiotics and −2.7 for BSAs (Table 3). University HE status strongly affected the results by exhibiting a high unique explanatory strength (4%–13%), particularly with the use of BDs as denominator.

### Physician and nurse staffing and the length of hospital stay

The variable describing the rate of physicians per hospital bed was found redundant in the regression analyses because of collinearity. By contrast, a change in the number of nurses per hospital bed correlated positively and strongly with all antibiotic use and all penicillin use, demonstrated by all four measurement units, but only marginally with BSA use measured with haDDDs. One unit change between HEs of nurse staffing rate (inter-HE range, 167.6 nurses/100 hospital beds, Table 2) was independently associated with a difference of 0.17 DDDs/100 BDs for all antibiotics and of 0.1 DDDs/100 BDs for all penicillins (Table 3).

A positive and moderate to strong correlation with antibiotic use was found for the two variables characterizing the length of hospital stay. The proportion of hospital stays of < 2 days was significantly correlated with increased antibiotic use in relation to 100 BDs (Table 3). In particular, the percentage of short hospital stays correlated strongly with high BSA use both for DDDs and for haDDDs, and the variable contributed 7% and 8% to the observed variances in the two models, respectively. A change of 1% in the proportion of short hospital stays resulted in a dose change of 0.3 for both indices. A high proportion of hospital stays > 10 days correlated positively and strongly with antibiotic use in all six models using number of discharges as the denominator (Table 4). This variable contributed significantly to the observed variances in these models, particularly for BSA use where the explanatory strength was as high as 8% and 12% for WHO DDDs and haDDDs, respectively.

### Diagnosis-related variables

Two variables related to the medical condition of the patients were correlated with antibiotic use. The proportion of patient hospital stays with an infectious disease main ICD-10 diagnosis was highly significant (P <0.01) and strongly correlated (beta weights of 0.36 to 0.47) with all antibiotic use and with the use of penicillins, but not with BSA use. One percent change in the proportion of ICD-10 infectious diseases main diagnosis was associated with a difference between two HEs in all antibiotic use of 5.35 DDDs/100 BDs and in penicillin use of 3.83 DDDs/100 BDs (see regression coefficients, Table 3). However, the interpretation of this finding should take into consideration that the interval of the observed data range was only 4.4% (Table 1), i.e. the range of unit differences between HEs for this explanatory variable was narrow. Also of note, ICD-10 infectious diseases main diagnosis contributed uniquely to 4%–7% of the observed variances in the eight models (increments in R^2^).

The proportion of hospital stays with surgical DRGs contributed 6% and 3% to the overall variances in the models for all antibiotics use, measured with haDDDs per 100 BDs and per 100 discharges, respectively. The proportion of surgical DRGs varied between HEs with an interval of 21.8% (Table 2) and each percentage difference between two HEs was associated with a change in all antibiotic use of 0.15 haDDDs/100 BDs and 0.51 haDDDs/100 discharges.

## Discussion

An increase in all antibiotic use and BSA use observed during the six-year study period has been reported in more detail [10]. We have also previously, by use of another data set, reported sizeable differences in antibiotic use between various Norwegian HEs [16].

In the present study, we identified several factors that were associated with dissimilarities in antibiotic use between HEs in Norway. High levels of nurse staffing, a high proportion of hospital stays with an infectious disease main ICD-10 diagnosis or a principal surgical DRG, and high proportions of short or long hospital stays were associated with increased antibiotic use. On the contrary, university affiliation was strongly associated with lower antibiotic use. The other hospital-associated variables—hospital size and geographical location—were not correlated with the levels of antibiotic use.

A main finding was the robustness that these variables exhibited across all models. Regardless of the measurement unit, with few exceptions the same explanatory variables were significantly related to antibiotic use within each of the three outcome (antibiotic) groups. Thus, in our opinion, the variables are valid for the evaluation of antibiotic surveillance results when different units of measurements are applied [9,11].

Our finding that the university HEs had significantly lower consumption of antibiotics than non-university hospitals in all models may seem surprising. For example, it contrasts the result of a German study of 145 acute care hospitals where regional variances in all antibiotic use were investigated [17]. Regional variances were not identified, but higher levels of use correlated significantly with hospital university affiliation. However, the German study did not include all specialties of the university hospitals but targeted only high-consumption units (surgical and internal medical wards, intensive care and haematology/oncology units). Contrary to this, our analyses were done on whole HEs because administrative data on the level of medical specialties were not available for the period in question.

The most probable explanation for the discrepancy is the relatively large subset in our university hospitals of units with a low usage of antibiotics, such as maternity/obstetric units, rehabilitation wards and paediatric specialties.

In support of this view is an additional analysis, which we undertook of a published data set for the period 2002–2007 [16]; this data set contained information about the various specialties. We found that the proportion of bed days in core units, out of all HE bed days, were significantly lower in university hospitals than in non-university hospitals (64% versus 79%; other data of the analysis not shown here). Furthermore, the use of antibiotics in core units was similar in university and non-university hospitals. Also, the antibiotic utilization level was almost four-fold higher in core units than in other units.

A French study also lends support to the explanation above [18]. In a study of 77 public hospitals, it was found that the relative number of patient days spent in internal medicine, surgical and intensive care units could explain most of the variability in antibiotic utilization. The authors concluded that there is a need to establish country-specific factors to aid interpretation of surveillance results. This is in accordance with our view that separate analyses should be carried out for university and non-university institutions.

Our finding of a strong positive correlation between high levels of nurse staffing and antibiotic use does not imply a causal relationship, but rather that high nurse ratios may be a surrogate marker for high proportions of severely ill patients [19]. In general, higher nurse staffing is related to higher levels of intensive care and more patients with complicated medical conditions.

Both shorter stays (<2 days) and longer stays (>10 days) were strongly associated with increased BSA use. It appears that the proportion of short hospital stays should be used as an adjustment factor when interpreting surveillance results using number of BDs as the denominator. By contrast, longer stays have an impact on the results when the number of discharges is the denominator. A possible explanation for the positive correlation between short stays and BSA use may be more extensive empiric antibiotic treatment on hospital admission, before culture results and other diagnostic results are available. The correlation to prolonged hospital stays may be explained by more frequent BSA use in patients treated for complicated conditions. That short hospital stays are linked to extensive utilization of antibiotics is consistent with the finding in an Israeli study where one-day hospitalizations were associated with high consumption of antibiotics [20].

The strong relationship between an infectious disease main ICD-10 diagnosis and all antibiotic and all penicillin use is plausible. The reason why this variable showed no independent association with BSA use may be related to a sparse data set, as use of BSAs in Norwegian hospitals, although exhibiting an alarming increase, is still limited [16]. In addition, in severely ill patients for whom BSAs are extensively used, a serious underlying condition rather than the superimposed infections tends to be registered as the main diagnosis.

A positive correlation between all antibiotic use and surgical DRGs may reflect a high consumption of antibiotics for preoperative prophylaxis and for treatment of postoperative infections. The finding that surgical services were associated with higher antibiotic use than medical services may seem unexpected since the latter are often considered more antibiotic-intensive units. However, this opinion could be challenged. Of note, a strong association was only found when haDDDs was applied, not with the use of WHO DDDs. In a previous study, we have shown that the use of haDDDs reflects hospital prescription recommendations better than WHO DDDs. Among other things, an underestimation of the proportion of metronidazole, cefalotin and doxycycline use, which appears when WHO DDDs are applied, is abrogated with use of haDDD. These drugs are used extensively – and cefalotin almost exclusively – for surgical prophylaxis in Norwegian hospitals [10,13]. Accordingly, more nuances are needed when comparing medical and surgical departments.

The lack of data on the distribution of medical specialties within the various HEs may be considered a limitation of our study. To request these administrative data from each HE would be a task not compatible with a routine national surveillance and, moreover, doubts might be raised with regard to the quality of locally obtained data. In addition, a correct allocation of patients to their specialties merely based on the wards designation is made difficult because of increasingly complex internal logistics in hospitals, mainly resulting from space limitations and task sharing between departments. However, in the future antibiotic data may be more reliantly linked to specialties through electronic prescribing modules integrated in patient-administrative systems.

Another potential limitation is the fact that we were not in possession of any specific parameter defining the severity of illness. A main challenge in the benchmarking of hospital performance, including the use of antibiotics, is to adjust for patient case-mix [21]. However, to date no case-mix model has been generally and unanimously endorsed. Moreover, certain limitations are present in studies investigating case-mix for limited time periods [22] or case-mix based on repeated prevalence surveys [23]. It should also be noted that the longitudinal studies discussed above [18,20] examined specific clinical variables and morbidity indices, such as the Charlson score [24], but none were found to be independently correlated with antibiotic use.

Finally, we have not considered any hospital data on antibiotic resistance. On a national scale, antibiotic resistance in hospital- or community-acquired pathogens is unlikely to be a major determinant for differences in antibiotic use. The reason for this is that the prevalence of resistance in Norwegian hospitals remains low [16,25].

A particular strength of our study is the inclusion of all Norwegian hospitals and the long observation period of six years. In addition, the independent variables investigated were based on official information from validated and easily available national sources, which makes them applicable for a routine surveillance system. To our knowledge, this is the first study to apply this kind of data set.

Furthermore, with regard to the statistical analyses we assessed the possible non-independence of repeated measures within HEs. The possibility of such non-independence made us introduce HEs as clusters in the regression models. However, the results obtained by use of this robust model were largely the same as those found with a standard multiple regression technique (data not shown).

Other outcome variables related to antibiotic utilization, for example health-care associated infections [26-28] and antibiotic-resistant infections [29], have been investigated for the purpose of adjusting for inter-hospital differences. Probably, these methods may be applicable also for benchmarking of hospital antibiotic use.

Although the factors identified in our study contributed substantially to differences in utilization of antibiotics in hospitals, there is still a sizable residual variance (30%–50% for all antibiotic use and 60%–70% for BSA use) that cannot be explained by these factors. Of these non-identified factors, attitudes and personal preferences of leading prescribing physicians are probably of special relevance [30]. Medical culture in general, levels of education, whether or not guidelines exist, and where they do, their content and quality, are elements that should be considered. Particular attention should be given to the use of BSAs because their use may lead to the development of resistant microbes. There is a need for heightened awareness with regard to the consequences of untoward use of these antibiotics.

Almost none of the explanatory factors demonstrated in this study are modifiable through interventions by health care workers or hospital administrators. However, it is crucial to reveal inappropriate antibiotic use and to establish prudent prescription behaviour [31,32]. Knowledge about existing non-modifiable conditions provides a much-needed in-depth understanding of antibiotic surveillance results. With this background knowledge, it becomes easier to identify which findings are related to inappropriate prescribing practices.

## Conclusions

While several strategies may be used to achieve a prudent use of antibiotics in hospitals [33], one initial step should be to identify the factors that are not prescriber related. Adjustments for non-modifiable factors, such as the ones we have established, may increase the confidence in observed surveillance results. This method thus enables us to better identify and target explicit areas for intervention measures.
